# Effectiveness of a Universal Parental Support Programme to Promote Healthy Dietary Habits and Physical Activity and to Prevent Overweight and Obesity in 6-Year-Old Children: The Healthy School Start Study, a Cluster-Randomised Controlled Trial

**DOI:** 10.1371/journal.pone.0116876

**Published:** 2015-02-13

**Authors:** Gisela Nyberg, Elinor Sundblom, Åsa Norman, Benjamin Bohman, Jan Hagberg, Liselotte Schäfer Elinder

**Affiliations:** 1 Karolinska Institutet, Department of Public Health Sciences, Tomtebodavägen 18 A, 171 65 Solna, Sweden; 2 Centre for Epidemiology and Community Medicine, Stockholm County Council, Box 1497, 171 29 Solna, Sweden; 3 Karolinska Institutet, Department of Clinical Neuroscience, Nobels väg 9, 171 65 Solna, Sweden; 4 Karolinska Institutet, Institute of Environmental Medicine, Box 210, 171 77 Stockholm, Sweden; University of Pennsylvania, UNITED STATES

## Abstract

**Objective:**

To develop and evaluate the effectiveness of a parental support programme to promote healthy dietary and physical activity habits and to prevent overweight and obesity in Swedish children.

**Methods:**

A cluster-randomised controlled trial was carried out in areas with low to medium socio-economic status. Participants were six-year-old children (n = 243) and their parents. Fourteen pre-school classes were randomly assigned to intervention (n = 7) and control groups (n = 7). The intervention lasted for 6 months and included: 1) Health information for parents, 2) Motivational Interviewing with parents and 3) Teacher-led classroom activities with children. Physical activity was measured by accelerometry, dietary and physical activity habits and parental self-efficacy through a questionnaire. Body weight and height were measured and BMI standard deviation score was calculated. Measurements were conducted at baseline, post-intervention and at 6-months follow-up. Group differences were examined using analysis of covariance and Poisson regression, adjusted for gender and baseline values.

**Results:**

There was no significant intervention effect in the primary outcome physical activity. Sub-group analyses showed a significant gender-group interaction in total physical activity (TPA), with girls in the intervention group demonstrating higher TPA during weekends (p = 0.04), as well as in sedentary time, with boys showing more sedentary time in the intervention group (p = 0.03). There was a significantly higher vegetable intake (0.26 servings) in the intervention group compared to the control group (p = 0.003). At follow-up, sub-group analyses showed a sustained effect for boys. The intervention did not affect the prevalence of overweight or obesity.

**Conclusions:**

It is possible to influence vegetable intake in children and girls’ physical activity through a parental support programme. The programme needs to be intensified in order to increase effectiveness and sustain the effects long-term. These findings are an important contribution to the further development of evidence-based parental support programmes to prevent overweight and obesity in children.

**Trial Registration:**

Controlled-trials.com ISRCTN32750699

## Introduction

Poor dietary habits and insufficient physical activity are among the most important modifiable lifestyle factors causing a substantial chronic disease burden worldwide including obesity [1]. There is evidence to suggest that health-related behaviours [2,3,4] and obesity [5] track to a certain degree from childhood to adolescence and adulthood, which may have serious health consequences in later life, such as metabolic disturbances, type 2 diabetes, cardiovascular diseases, certain cancers and impaired mobility [6].

The health of children in Sweden is good by international standards, but there are large social inequalities in dietary habits, physical activity and prevalence of obesity [7,8]. For example, the prevalence of obesity in 10-year-old children in deprived areas is three times higher than in affluent areas, pointing to the importance of social factors [7].

Parents are important for influencing children’s dietary and physical activity habits. Factors in the home environment that are important for children’s dietary habits are availability of and accessibility to healthy foods at home, parents’ own dietary habits, parenting styles and parental feeding styles and practices [9,10,11,12]. Correlates of importance for physical activity levels in children are parental support, encouragement, doing activities with parents and parents’ own physical activity levels [13,14,15,16].

There is mounting evidence that schools are effective settings for promoting healthy eating habits and physical activity of children and prevention of unhealthy weight gain, although the effects achieved are usually small and short-lived [17,18,19,20]. Parental involvement however, leads to a higher intervention effect, which has been seen in studies on prevention of overweight and obesity [19,20,21,22]. Waters and colleagues concluded that promising strategies, among others, included parent support and home activities that encourage children to be more active, eat more nutritious foods and spend less time on screen based activities. Other reviews regarding parental involvement concluded that programmes that target parents indirectly, for example through newsletters, have less effect compared to programmes targeting parents directly e.g. where parents’ participation is required [23,24]. Golley et al. summarised the literature regarding the effectiveness of interventions that involve parents to improve children’s weight-related behaviours and concluded that effective interventions had similar features: better study quality, parents responsible for participation and implementation, greater parental involvement and inclusion of prompt barrier identification, restructuring of the home environment, prompt self-monitoring, and use of prompt specific goal setting behaviour change techniques [25]. With regard to physical activity in particular, counselling with parents shows some promise in promoting physical activity in children, although the evidence is weak due to heterogeneity of study design, study quality and outcome measures [24].

It is widely recommended that the programme design should be based on theory [26]. A theoretical framework supports the identification of possible mediators. Social cognitive theory (SCT) explains behaviour as a reciprocal interaction between person, behaviour, and environmental factors [27,28]. A central construct in SCT is self-efficacy, a person’s belief in his or her ability to successfully perform a certain action. Young children have a limited cognitive capacity to make such self-judgments and are therefore strongly dependent on external factors provided by others in the home and in school [29,30,31,32], for example, access to healthy food and opportunities to be physically active.

One way to improve parental self-efficacy (PSE) could be through Motivational Interviewing (MI), which is a method used to support behaviour change. MI is a client-centred, goal-oriented communication style designed to strengthen personal motivation for a specific behaviour change [33]. This is achieved through the exploration of thoughts, values and emotions regarding the change. The counsellor provides support by evoking arguments from the person, with a focus on statements which are positive towards the change. MI also focuses on the person´s own belief in his or her ability to succeed with a behaviour change, hence supporting the person´s self-efficacy. In the treatment and prevention of overweight and obesity, evidence has been found that the use of MI may lead to improved dietary and physical activity habits in adults [34] and enhanced weight loss in overweight and obese patients [35]. Little is yet known of the effectiveness of MI in influencing parents with the aim to affect the lifestyle of their children, but one study indicated a positive impact [36]. The main intervention component in this study was MI, which was hypothesised to influence parental self-efficacy as a mediator to provide support for healthy eating and physical activity in children. Only a few studies have investigated the associations between parental self-efficacy and children’s diet [29,30,37], physical activity [31,37] and sedentary behaviours [30,37].

The aim of this study was to evaluate the effectiveness of the 6-month Healthy School Start programme on children’s physical activity and healthy eating habits and on the prevention of overweight and obesity in six-year-old children attending pre-school class.

## Methods

The protocol for this trial and supporting CONSORT checklist are available as supporting information; see S1 Checklist and S1 Protocol.

### Study design, setting and participants

The Healthy School Start study was designed as a cluster-randomised controlled trial with a waiting list control group. The unit of randomisation was pre-school class. The study protocol has been published [39]. Schools were chosen from a municipality in Stockholm County, Sweden, with a population of low to medium socio-economic status (SES) and with mixed types of housing (blocks of flats, semi-detached houses and detached houses). The schools included were within the school physician’s administrational area.

**Figure 1:**
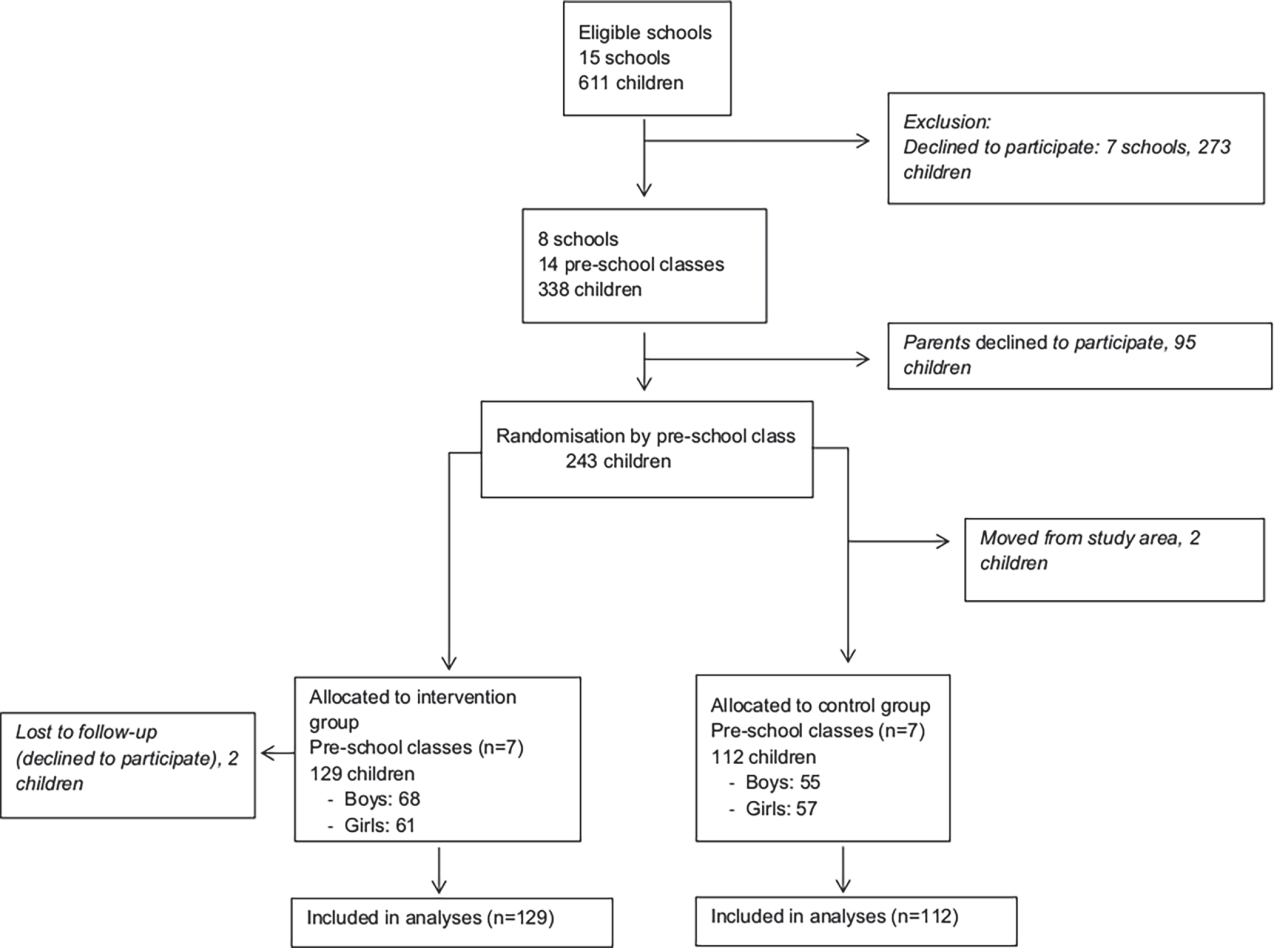
Flow diagram of participants.

Fig. 1 shows a flow chart of participant recruitment and retention. There were 15 eligible schools (n = 611 children) in the administrational area. Seven schools declined to participate mainly due to time constraint. In total, eight schools were included and all the 14 pre-school classes in these schools participated. All families who had children in these pre-school classes were invited to participate in the study, provided that at least one parent was able to communicate and understand the Swedish language. Of the 338 children in the study, 243 agreed to participate (72%). The classes were randomly assigned by the research team to intervention (n = 7) and waiting-list control groups (n = 7). The children were recruited in August to September 2010 and the intervention started in October and lasted for six months during pre-school class (2010–2011).

Reasons for choosing pre-school class include the fact that this stage marks a new period in children’s lives, when parents are eager to give them a good start and the children are still very much under the influence of their parents. Furthermore, during the pre-school year teachers do not have a strict curriculum to follow and therefore have greater flexibility in the classroom, and health issues like diet and physical activity fit very well with the educational objectives at this stage. Pre-school class is not compulsory in Sweden but more than 95 percent of all six-year-old children attend. All children receive a free hot lunch every day in school.

Written consent was collected from all parents of participating children. Ethical permission was approved from the Regional Ethical Review Board in Stockholm, Sweden (2010/934–31/1) on the 23^rd^ of June, 2010. The authors confirm that all ongoing and related trials for this intervention are registered.

Trial registration: ISRCTN32750699. The development of the MI intervention component was not completely finished before the enrolment of participants started and therefore there was a delay in registering the study. The trial was registered in December 2010.

### Theory

A problem theory and a programme theory were developed [40]. The problem theory was based on SCT [28]. In the context of this intervention, parental knowledge, attitude, preference, care and control, role modelling, willingness to change and parental self-efficacy were identified as possible mediators of children’s dietary and physical activity habits and weight development. Based on this analysis, the programme theory was developed, with three components to target the identified mediators.

### Intervention components

The programme was comprised of three components:

Health information

A brochure was developed with the aim to increase parental knowledge on how to promote children’s dietary and physical activity habits based on a literature review [41]. The brochure contains facts and advice for parents within seven areas: 1) parental feeding practices; 2) healthy food and family meal times; 3) physical activity; 4) sweets, snacks, ice-cream and sodas; 5) fruit and vegetables; 6) physical inactivity, screen time, and commercials; 7) sleep. The brochure was sent home to parents.

Motivational interviewing

MI was used to target and increase parental care and control and self-efficacy to provide support for healthy eating and physical activity to the child, as well as to stimulate willingness to change. Parents in the intervention group were offered two sessions of MI during the intervention period with a trained person performing MI (MI-counsellor), without the presence of the child. Each session lasted 45 minutes where the parents, with the help of the brochure, had the opportunity to choose and focus on a specific change regarding their child’s dietary habits or physical activity. In line with MI, the parents were supported in focusing, goal-setting and planning as well as assisted by the MI counsellor in the exploration of personal values and the advantages of a change. During the first MI-session, the parents chose a target behaviour regarding their child´s diet or physical activity that they wanted to alter or change, using an agenda-setting tool. The target behaviour was subsequently explored together with the MI-counsellor and the parent got an opportunity to set a goal related to the focus area to work on at home until the second MI session, which served as a follow-up.

Classroom activities

The classroom component was based on a literature review and was inspired by earlier school interventions [41,42,43], with the aim to target the children’s knowledge, attitudes and preferences and the parents’ role modelling for healthy behaviours. A teacher’s manual and a workbook for children were developed to facilitate the classroom activities. The activities were related to the different areas in the brochure, for example discussing the importance of eating fruit and vegetables and thereafter trying a new fruit or vegetable. The materials were developed together with teachers and pre-tested in a group of six-year-old children and their parents. The children were exposed to ten 30-minute teacher-led sessions. The teachers were provided with a tool-box and used the teaching manual for each session. After most sessions the children were given homework in their workbooks with the aim to discuss the session and perform related activities at home with their parents/guardians. Back in the class room, the teachers and children summarised the homework, so that each theme was repeated together with the children.

Control classes were offered the whole programme directly after the 6-months follow-up measurements.

### Implementation strategies

The following implementation strategies were used:

Schools

The principals in each school signed an agreement with the research group specifying the obligations and commitments of the schools and the research team.

Teachers

The teachers were trained for the classroom activities by the research team for two hours. Also, teachers were given continuous support by the research team through face-to face contact and by telephone and e-mail throughout the intervention.

Parents

Parents were informed about the project at the first regular information meeting in school.

### Outcome evaluation

Data was collected at baseline between August and September 2010 (time 1), directly after the intervention between April and May 2011 (time 2) and at follow-up six months after the intervention, between September and October 2011, (time 3).

### Primary outcomes

Physical activity by accelerometry

Physical activity was measured by accelerometry (GT3 XP, Actigraph, LCC, Pensacola, USA) for seven consecutive days. The Actigraph has been found to be a valid and reliable method for measuring physical activity in children and has been used in many studies [44,45].

Accelerometry data were analysed with the Actilife (Actigraph) software (version 6.5.3.). Physical activity variables include total physical activity (TPA), time spent in moderate to vigorous physical activity (MVPA) intensity and time spent in sedentary intensity, averaged per day over the assessment period. Physical activity was assessed between 8 am and 9 pm and was calculated for the whole week and during weekends. Children who provided at least 600 minutes of activity registration per day for a minimum of 3 days were included in the analyses. Data was defined as non-wear time and was excluded if sequences showed 10 or more consecutive zero counts. The epoch length was set to 15 seconds. Thresholds were set to categorise time in sedentary (≤ 149 cpm) and moderate to vigorous (≥ 500 cpm) intensity [46].

Health behaviours by parent report

Indicator foods (fruit, vegetables and energy-dense products), physical activity habits, sedentary behaviour and sleep were measured through a validated parent-proxy questionnaire, the Eating and Physical Activity Questionnaire (EPAQ) [47], which was translated into Swedish and to some extent adapted to the Swedish context. The EPAQ is a questionnaire assessing the previous day’s dietary intake in mean servings of food and beverages. The questionnaire has been validated against 24-hour dietary recall in 2 to 5-year-old children and showed significant Spearman rank correlations for different items ranging from 0.57 to 0.88 [47]. The EPAQ was distributed by mail to the parents.

### Secondary outcomes

Self-efficacy by self-report

Parental self-efficacy was measured through a questionnaire developed by the authors in response to a lack of such a measure at the time of commencing the study. Items were developed by the authors in accordance with guidelines for self-efficacy item wording and scale construction [48], and by reviewing previous PSE measures, in particular the Tool to Measure Parenting Self-Efficacy (TOPSE) [49]. Validity and reliability of our scale has been assessed and reported by our group [37]. Factor analysis showed that a 14-item version of the scale was composed of the following three factors of PSE regarding dietary and physical activity behaviours in children: PSE for controlling unhealthy dietary and physical activity behaviours in children (six items), PSE for engaging children in healthy physical activity behaviours (five items), and PSE for arranging a positive meal pattern for children (three items). The questionnaire was distributed by mail to the parents, together with the EPAQ.

Anthropometry

Height, weight and waist circumference measurements were performed in schools by two trained research assistants according to standardised procedures [39]. BMI was calculated as weight (kg) divided by height (m) squared. Overweight and obesity were defined according to the International Obesity Task Force recommendations [50]. BMI standard deviation score (BMIsds) was calculated according to a Swedish reference standard [51].

### Socio-economic status

The highest level of parental education for the mother or father was used as an indicator of SES and was divided into low (4 years or less of upper secondary school) or high (more than 4 years of upper secondary school).

### Process evaluation

Fidelity to the three intervention components was measured through coding of MI sessions and recording of activities. The parents were asked at the first MI session if they had read the brochure. Compliance with the teaching sessions and workbook completion was monitored by questions and by continuous contact with the teachers. The teachers documented in a check list how much time they had spent on each session.

MI sessions were audio-recorded and subsequently coded for MI behaviour according to the Motivational Interviewing Treatment Integrity (MITI) Code [52]. Ten percent of the sessions were recorded and coded (19 sessions). MITI coding includes several different variables of MI adherent and MI non-adherent practitioner behaviour. We chose to present “MI Spirit” because this variable is central to the use of MI and has proven to be the most difficult to learn [53]. The threshold for beginners’ proficiency in “MI Spirit” has been set to an average of 3.5, in accordance with expert opinion [52].

### Data analyses

Data analyses were performed using IBM SPSS Statistics (version 20 for Windows, 2011, SPSS Inc., Chicago, IL). The level of statistical significance was set at p<0.05. Descriptive statistics are presented as means and standard deviations. Independent samples t-test was used for continuous data and chi square test for categorical data to test for group differences at baseline. For continuous variables, differences between intervention and control groups in physical activity (TPA, MVPA and sedentary), PSE, and BMIsds were analysed using analysis of covariance (ANCOVA), including sex as a fixed factor and baseline value as a covariate. Additionally, ANCOVA analyses with physical activity outcomes were adjusted for monitor wear time as a covariate. In the initial ANCOVA analyses, models were adjusted for SES defined as parental education as a fixed factor and analyses were performed with correction for cluster variation by including a random intercept to estimate variation in outcomes between the pre-school classes. Neither SES nor variation between pre-school class was significant and adjustments were therefore not made in the final models. To evaluate the effect on health behaviours expressed as categorical data in the form of counts, (servings per day of fruit juice, soft drink, milk, flavoured milk, vegetables, snacks, fruit, sweets, cookies and ice-cream), Generalised Linear Models were used to conduct Poisson regressions including sex as a fixed factor and baseline value as a covariate. To evaluate the effects on health behaviours with continuous data (usual servings of vegetables per day, child taken to activity in the last week and screen time viewing) ANCOVA was used including sex as a fixed factor and baseline value as a covariate. When an interaction effect was found for group × sex in the ANCOVA analyses and in the Poisson regression analyses, the analyses were stratified on sex.

The assumptions for the statistical tests were fulfilled. The continuous outcomes in the ANCOVA analyses were normally distributed and had equal variances between groups (homogeneity) for each outcome. In the Poisson regression for categorical outcomes the assumptions regarding over-dispersion and multicollinearity of predictors were fulfilled. Results for physical activity and the other health behaviours are expressed as beta values. Positive values denote a higher value in the intervention group. Results are expressed as means with 95% confidence intervals and corresponding p-values.

Our main priority was to examine potential intervention effects before and after the intervention (time 1 and time 2) and then to see if the effect was maintained at follow-up (time 3). Therefore, outcomes at time 1 were first compared to time 2 and then to time 3. For the follow-up analyses (time 3 compared to time 1), only those children were included who also had data in time 2 in order to ensure comparability.

The primary analysis was carried out using the observed cases population and an intention to treat analysis was performed with replacement for missing data using the last value carried forward approach.

The power calculation for this study was based on the assumption of an average 20% increase of physical activity assessed by accelerometry in the intervention group. The estimated sample size was calculated for a two-sided test with the significance level of 0.05 and power was set to 90% using a sample size calculator for cluster randomized trials [38]. The calculation showed that 12 school classes with a participation rate of 60% in each class, approximately 144 children in total, were needed to detect a 20% change in physical activity between the intervention and the control group. It was decided to randomise 7+7 pre-school classes with approximately 100 children in intervention group and 100 children in control group to have a margin for attrition. Regarding dietary outcomes, relevant data on intake was not available at that time point to do a power calculation.

## Results

In total, four children dropped out of the study (intervention: n = 2, control: n = 2). There were no differences in age and anthropometric measures at baseline between intervention and control groups as shown in Table 1. Thirty-three percent (n = 41) of the parents in the intervention group and 40% (n = 40) in the control group had a low level of education with no significant difference among the groups. In total, 70% of the parents were born in Sweden, 7% in Europe and 23% were born outside of Europe.

**Table 1:** Descriptive characteristics of children at baseline categorised by intervention and control group.

	n	Total n = 241	Intervention n = 129 (68 boys/61 girls)	Control n = 112 (55 boys/57 girls)	p
		Mean (SD)	Mean (SD)	Mean (SD)	
Age (years)	241	6.2 (0.3)	6.2 (0.3)	6.2 (0.3)	0.30
Anthropometry					
Weight (kg)	241	23.4 (4.1)	23.3 (3.7)	23.6 (4.6)	0.51
Height (cm)	241	119.5 (5.5)	119.8 (5.6)	119.0 (5.5)	0.26
Waist circumference (cm)	241	56.6 (5.2)	56.4 (4.7)	56.9 (5.8)	0.45
Body mass index (kg/m2)	241	16.3 (2.0)	16.1 (1.8)	16.6 (2.2)	0.11
BMIsds	241	0.41 (1.19)	0.29 (1.13)	0.55 (1.24)	0.09
Normal weight (%)	241	77.2	80.6	73.2	0.32
Overweight and obese (%)	241	19.1	14.7	24.1	0.21
Underweight (%)	241	3.7	4.7	2.7	0.79
Physical activity					
TPA, all week (cpm)	195	747 (175)	769 (186)	720 (158)	0.053
TPA, weekend (cpm)	148	659 (215)	688 (248)	619 (150)	0.055
MVPA, all week (minutes)	195	242 (39)	240 (39)	246 (38)	0.26
MVPA, weekend (minutes)	148	218 (52)	214 (56)	222 (46)	0.38
Sedentary, all week (percent)	195	0.50 (0.06)	0.51 (0.05)	0.49 (0.06)	0.07
Sedentary, weekends (percent)	148	0.53 (0.08)	0.54 (0.08)	0.52 (0.07)	0.22
Behaviour					
Fruit juice (servings the previous day)	195	0.57 (0.81)	0.59 (0.90)	0.54 (0.68)	0.67
Soft drink (servings the previous day)	175	0.19 (0.46)	0.12 (0.36)	0.28 (0.56)	0.02
Milk (servings the previous day)	210	1.73 (1.07)	1.82 (1.12)	1.62 (1.00)	0.20
Flavoured milk (servings the previous day)	164	0.23 (0.43)	0.16 (0.37)	0.30 (0.49)	0.03
Vegetables (servings the previous day)	220	1.43 (1.08)	1.38 (1.06)	1.48 (1.11)	0.50
Fruits (servings the previous day)	224	2.29 (1.31)	2.29 (1.37)	2.28 (1.23)	0.93
Snacks (servings the previous day)	200	0.13 (0.60)	0.11 (0.64)	0.15 (0.54)	0.64
Chocolate/candy (servings the previous day)	200	0.30 (0.84)	0.28 (0.85)	0.32 (0.83)	0.75
Ice cream (servings the previous day)	205	0.46 (0.91)	0.41 (0.84)	0.51 (1.00)	0.42
Cake/buns/cookies (servings the previous day)	199	0.41 (0.84)	0.41 (0.80)	0.42 (0.89)	0.92
Usual servings of vegetables per day	223	1.12 (0.67)	1.08 (0.62)	1.16 (0.73)	0.38
Child taken to playground etc in the					
last week (times/week)	224	2.5 (1.48)	2.7 (1.4)	2.3 (1.6)	0.08
Television/computer time (minutes/day)	218	94 (61)	90 (57)	98 (65)	0.33
Parental self-efficacy (PSE)					
Factor 1^a^	229	57.3 (9.5)	58.0 (9.4)	56.4 (9.7)	0.22
Factor 2^b^	229	39.8 (6.7)	40.3 (6.3)	39.3 (7.1)	0.21
Factor 3^c^	229	24.7 (4.2)	24.8 (4.0)	24.6 (4.4)	0.70
Total sum	223	113.5 (16.2)	114.4 (15.8)	112.2 (16.7)	0.33

Data are means and standard deviations (SD).

p = between intervention and control groups, TPA: total physical activity, cpm = counts per minute, MVPA: moderate to vigorous physical activity.

Results of independent t-test and chi-square test.

^a^PSE for controlling unhealthy dietary and PA behaviours in children.

^b^PSE for engaging children in healthy PA behaviours.

^c^PSE for arranging positive meal patterns for children.

### Physical activity

A total of 195 children (81%) had a minimum of 3 valid days of accelerometer data at baseline, 186 children (77%) at time 2 and 185 children (77%) at time 3.

All the 241 children (100%) reached 60 minutes of MVPA at baseline.

After the intervention, at time 2, there were no significant differences between the intervention and control groups in physical activity (TPA, MVPA and time spent sedentary), neither for the whole week nor for the weekend (Table 2). An interaction effect was found for sex (group × sex), showing a significant intervention effect with higher TPA (cpm) during weekends for girls compared to the girls in the control group (p = 0.04, 95% CI = 13.09 to 436.0). Girls in the intervention group had a mean value of 225 cpm higher TPA compared to the girls in the control group. In addition, a significant interaction effect with sex (group × sex) was found for sedentary time for the whole week for boys (p = 0.03, 95% CI = 0.29 to 5.02). After the intervention, boys in the intervention group had significantly higher sedentary time compared to the boys in the control group (2.7% of total wear time). However, at time 3 there were no longer any significant differences in any of the physical activity variables or sedentary time between the intervention and the control groups.

**Table 2:** Effects of the intervention on physical activity levels at time 2 and time 3.

		Time 2				Time 3		
	n	b	95% CI	p	n	b	95% CI	p
TPA, all week (cpm)	156	-21.2	-96.5 to 54.0	0.58	138	-15.0	-60.2 to 30.3	0.51
TPA, weekends (cpm)	105	94.4	-45.0 to 233.8	0.18	81	65.1	-101.7 to 231.9	0.44
TPA, weekends, girls (cpm)^a^	56	225	13.1 to 436	0.04				
TPA, weekends, boys (cpm)^a^	49	-47.0	-224.6 to 130.6	0.60				
MVPA, all week (minutes)	156	-4.9	-14.7 to 5.0	0.33	138	2.7	-7.5 to 12.9	0.60
MVPA, weekends (minutes)	105	-3.8	-26.0 to 18.3	0.73	105	16.8	-8.5 to 42.0	0.19
Sedentary, all week (%)	156	0.4	-1.1 to 2.0	0.59	138	-0.8	-2.2 to 0.6	0.27
Sedentary, all week, girls (%)^a^	81	-1.1	-3.1 to 0.9	0.26				
Sedentary, all week, boys (%)^a^	75	2.7	0.29 to 5.0	0.03				
Sedentary, weekends (%)	105	0.002	-3.2 to 3.2	1.00	81	-2.9	-7.1 to 1.2	0.16

b = Standardised regression coefficient (beta), CI = 95% confidence interval.

p = between intervention and control groups.

TPA: total physical activity, cpm: counts per minute, MVPA: moderate to vigorous physical activity.

Results of ANCOVA adjusted for sex, monitor wear time and baseline values.

^a^ stratified analysis due to interaction effect (group × sex) at time 2.

### Health behaviours

The response rate of parents answering the questionnaire measuring dietary and physical activity habits ranged between 68% and 93% for the different questions.

At time 2 there was a significantly higher number of “servings of vegetables usually eaten each day” in the intervention group compared to the control group (p = 0.003) showing a higher mean value of 0.26 servings of vegetables in the intervention group (Table 3). At time 3 there was no difference between the groups. However, at time 3 a significant interaction effect for sex (group × sex) was found for boys in the intervention group, showing a higher mean value of 0.39 servings of vegetables compared to boys in the control group, (p = 0.01, 95% CI = 0.09 to 0.69). An intention to treat analysis was performed for the variable “servings of vegetables usually eaten each day”. The significant difference between the intervention and control groups at time 2 remained significant (p = 0.002) as did the significant interaction effect at time 3 (p = 0.02). At times 2 and 3 there were no differences between the intervention and control groups in the other variables measuring health behaviours (fruit and energy-dense products), physical activity habits, sedentary behaviour or sleep (Table 3).

**Table 3:** Effects of the intervention on health related behaviours at time 2 and time 3.

		Time 2				Time 3		
	n	b	95% CI	p	n	b	95% CI	p
Behaviour								
Servings the previous weekday^a^								
Fruit juice^1^	148	-0.20	-0.64 to 0.24	0.38	115	-0.21	-0.72 to 0.29	0.41
Soft drink/sugar syrup^1^	134	-0.37	-0.99 to 0.24	0.23	103	0.20	-0.61 to 1.01	0.63
Milk^1^	175	0.04	-0.19 to 0.27	0.71	149	-0.01	-0.25 to 0.24	0.95
Flavoured milk^1^	117	0.04	-0.66 to 0.74	0.92	94	-0.18	-0.99 to 0.63	0.67
Vegetables^1^	188	0.09	-0.14 to 0.31	0.44	170	0.05	-0.19 to 0.29	0.67
Snacks^1^	171	-0.28	0.43 to 0.60	0.44	143	-0.67	-1.94 to 0.59	0.30
Fruit^1^	193	0.11	-0.8 to 0.30	0.26	169	0.13	-0.08 to 0.33	0.23
Sweets^1^	167	-0.003	-0.67 to 0.66	0.99	141	0.49	-0.31 to 1.30	0.23
Cakes/buns/cookies^1^	156	-0.25	-0.67 to 0.17	0.24	131	0.38	-0.30 to 1.00	0.24
Ice-cream^1^	178	0.08	-0.30 to 0.46	0.69	147	0.41	-0.19 to 1.01	0.18
Usual servings of								
vegetables per day^2^	198	0.26	0.09 to 0.43	0.003	183	0.14	-0.04 to 0.32	0.14
boys^b^					89	0.39	0.09 to 0.69	0.01
girls^b^					94	-0.1	-0.3 to 0.1	0.34
Child taken to activity								
in the last week (times/wk)^2^	192	-0.48	-0.10 to 0.03	0.07	172	-0.27	-0.69 to 0.16	0.22
Screen time viewing								
(min/day)^2^	180	-3.59	-26.18 to 19.00	0.76	166	-8.23	-23.66 to 7.20	0.29

b = Standardised regression coefficient (beta), CI = 95% confidence interval.

p = between intervention and control groups.

^1^Results of Generalised linear models regression analysis (Poisson distribution) adjusted for sex and baseline values.

^2^Results of ANCOVA adjusted for sex and baseline values.

^a^ serving sizes (examples below):

drinks = 1,5 dl

vegetables = 2 dl grated carrots/cabbage or a big tomato or 2–3 broccoli stalks

Fruit = a small apple or a bunch of grapes (about 10)

Snacks = 1,5 dl

Sweets = about 1,5 dl of sweets or 4 pieces from a of chocolate bar

Cakes = a small bun, or 5 small biscuits

Ice-cream = a small popsicle stick or 1 dl ice cream

^b^ stratified analysis due to interaction effect (group × sex) at time 3.

### Weight prevalence and weight development

Data on weight and height were collected for all children at baseline. Data for seven children were missing at time 2. All of these children were categorised as normal weight at baseline.

There were no significant differences between intervention and control groups in terms of change of prevalence of underweight, normal weight, overweight and obesity between time 2 and 1 and between time 3 and 1, as shown in Table 4.

**Table 4:** Prevalence of underweight, normal weight, overweight and obesity at time 1, time 2 and time 3.

	Time 1		Time 2		Time 3		Difference	p	Difference	p
	Int	Con	Int	Con	Int	Con	(I_2_-I_1_)-(C_2_-C_1_)		(I_3_-I_1_)-(C_3_-C_1_)	
	n = 124	n = 110	n = 124	n = 110	n = 123	n = 106				
Underweight (%)^§^	4.0	2.7	6.5	3.6	3.2	2.7	1.6	0.53	-0.8	0.69
Normal weight (%)^§^	80.6	72.7	81.5	75.5	80.6	71.8	-1.9	0.65	0.9	0.61
Overweight (%)^§^	9.7	17.3	6.5	11.8	8.9	11.8	2.3	0.54	4.7	0.43
Obese (%)^§^	5.6	7.3	5.6	9.1	6.5	10.0	-1.8	0.16	-1.8	0.37

p = between intervention and control groups.

§, defined according to Cole et al. 2012.

I_1_, Intervention group, time 1.

C_1_, Control group, time 1.

Results of independent t-test.

After the intervention, there was no significant difference between the intervention and control groups in BMIsds at time 2 and 3. The change between time 2 and 1 in BMIsds was-0.11 in the intervention group, compared to-0.06 in the control group. An analysis stratified for weight status showed a change between time 2 and 1 of-0.27 BMIsds in the sub-group of overweight and obese children in the intervention group, compared to-0.03 in the control group. This difference was not statistically significant.

### Parental self-efficacy

In total, 229 parents answered the questions on PSE at baseline. There were no differences in the total PSE score or the three factors at baseline between intervention and control groups. At time 2 and 3 there were also no differences between the intervention and control groups in the PSE factors or in the total sum of PSE.

### Process evaluation

Evaluation of fidelity showed that all the parents reported that they had read the brochure. For the classroom activities, the teachers spent on average 33 minutes on each lesson (range = 15–60 minutes). Three intervention classes did 10 lessons, three classes did 8 lessons and one class did 7 lessons. During the intervention, the first MI session was conducted with 110 parents. Of these 66 were mothers, 32 fathers and 12 parents came as a couple to the sessions. In the second MI session 89 of the initial 110 parents participated. During the first session just over 30% of the sessions were rescheduled at the parents’ request. The second session was rescheduled in as many as 60% of the occasions. During the MI sessions approximately two thirds of the parents were thorough in expressing that the focus area did not constitute a major problem in the family, but was merely a minor matter. The average of “MI Spirit variables”, measured with MITI version 3.1, was 3.52.

The implementation of the programme has been evaluated qualitatively. This evaluation showed that the programme was appreciated by teachers and parents and perceived as flexible and easy to implement (unpublished data).

## Discussion

This study evaluated the effectiveness of the Healthy School Start parental support programme to promote healthy dietary habits and physical activity in children. The results of this study show that there was no intervention effect on the whole group on physical activity but there was an effect post-intervention on girls’ total physical activity during weekends. An intervention effect was found for children’s intake of vegetables. However, this effect was only sustained for boys at 6-months follow-up.

### Physical activity and sedentary behaviour

In this study we did not find a significant intervention effect on total physical activity, time spent in MVPA or time spent sedentary between the intervention and control groups as a whole. One explanation for the lack of main intervention effect could be that the children in our study had high physical activity levels at baseline as all children fulfilled the recommendations of 60 minutes of MVPA per day. A positive effect was only seen in girls at weekends when activity levels were the lowest. In general, girls are less physically active than boys and a previous Swedish study showed that children are less active during weekends compared to weekdays [54]. It is encouraging that the changes in physical activity in our study concerned the gender most at risk for low physical activity. Several previous studies have also shown a stronger intervention effect for physical activity in girls than boys [55,56]. Metcalf and colleagues [57] reviewed the literature concerning physical activity interventions, measured objectively, on TPA and time spent in MVPA in children up to the age of 16 years. The authors came to the conclusion that interventions to increase physical activity only led to small improvements in MVPA, which corresponded to approximately 4 more minutes of walking or running per day. They concluded that the intervention effect for TPA was small to negligible, which is consistent with another review exploring the effect of physical activity interventions, showing only small but statistically significant increases in physical activity [58]. However, other reviews have reported positive effects of interventions on physical activity levels in children aged 6–18 years [18,59], but they included studies with both subjective and objective measurement of physical activity, which might partly explain the contrasting results. Further, a recent review found that physical activity interventions in children and adolescents with a duration longer than 1 year and based on theory were effective in sustaining an impact at follow-up ranging from 6 months to 20 years [60].

A programme [61] similar to ours with parental involvement was conducted in the UK during 10 months, with the aim to promote healthy lifestyles in 7 to 11-year-old children (n = 589). The programme was school-based, included activities performed by teachers and involved parents through homework tasks and by providing them with information. In contrast to our study, a significant increase in MVPA for the whole group was demonstrated, with 9 more minutes of MVPA per day in the intervention group but the programme showed no effect on fruit and vegetable intake. The study was a non-randomised controlled study, the children were slightly older and had lower levels of MVPA at baseline, 120 minutes, compared to our children, who had 242 minutes of MVPA, which could partly explain the difference in results. Further, in our study, boys in the intervention group were more sedentary after the intervention, showing that the intervention was not effective in decreasing sedentary behaviour. Thus, different strategies may be needed to target sedentary behaviours.

### Dietary behaviour

The results from our study showed a significantly higher intake of vegetables (0.26 portions/day) in the intervention group compared to the control group, and at follow-up the intervention effect was sustained for boys in the intervention group. These results are very encouraging since intake of fruit and vegetables is of great importance for health and shows a large gradient with regard to gender and socio-economic status, with boys and lower socio-economic groups having lower intakes [9]. It is therefore hopeful that our programme seems to have been successful in changing vegetable intake and for sustaining the effect in boys 6 months after the intervention ended. A similar effect on vegetable intake has been reported in an intervention in children up to 5 years [62]. The authors found that the vegetable intake was 0.13 portions higher in the intervention group compared to the control group. Further, in a recent meta-analysis the effect from school-based interventions in 5 to 12-year-old children (n = 26 361), showed an improvement in vegetable intake of 0.07 portions [63]. Multi-component programmes that involved both children and parents were more effective in improving fruit and vegetable intake compared to single-component programmes providing free or subsidised fruit and vegetables [63]. A long-term improvement of fruit and vegetable intake after two years has also been shown in a group of overweight children (n = 160) after a 6-month parental support intervention [64]. In comparison to our results, a study from Germany, with 5 to 6-year-old children (n = 2658), performed an intervention in day-care where they involved parents, with the aim to reduce obesity risk factors by modifying food habits [42]. The intervention led to an increased proportion of children with high fruit and vegetable consumption, and like in our study, the intervention showed an effect already after six months, with sustainable effects at a 5-months-follow-up. In contrast to our study, however, the authors did not find any differential effect in girls and boys.

### BMI

The results in our study showed no difference between the intervention and control groups for weight status and weight development after the intervention. However, a lower BMIsds was found in the group of children with overweight and obesity in the intervention group (-0.27 BMIsds) compared to the control group (-0.03), although this difference was not statistically significant. These are trends that need further investigation in future research. One reason for not detecting any significant differences regarding weight could be that the study was not powered for this. Results of studies of preventing overweight and obesity in children range from no effects to significant intervention effects on BMI [20,26]. This could partly be due to differences in intervention components, in age groups targeted and in the prevalence of overweight and obesity levels at baseline in the studied populations. Similar to our study, parental support was included in an intervention study with 9-year-old children (n = 209) from Italy [65]. The duration of the intervention was 5 months and the intervention components consisted of Motivational Interviewing with parents, extra physical activity in school and activities for both children and parents, including group discussions of dietary habits and physical activity. In contrast to our study, a significant difference between the intervention (-0.06 BMIsds) and the control group (0.12 BMIsds) was found. The change in the intervention group is of similar magnitude to the effect observed in our study in the intervention group (-0.11), but in our study even the control group showed a decrease in BMIsds (-0.06).

### Parental self-efficacy

Parental self-efficacy did not increase after the intervention. One reason for not seeing any effect could be that the responses were all in the high end of the scale at baseline and therefore difficult to increase. Another possibility could be that there are other mediators that are more important for changing physical activity and dietary behaviours in interventions, such as role modeling [66], parental knowledge and attitudes [67], parenting style and family functioning [68], which should be investigated in future studies.

### Motivational interviewing

During the MI sessions most parents emphasised that the focus area did not constitute a major problem in the family, but was merely a minor matter. Additionally, few parents chose a new focus area during the second session and these sessions were frequently rescheduled. This could indicate that, in a prevention programme, one MI session is enough for parents who do not perceive a major problem with their child´s health behaviours, whereas for other families several sessions may be required. Rescheduling of the MI sessions was frequent during the intervention and often happened shortly before the session making it difficult or impossible to meet another parent at the time of the cancelled appointment. This in turn resulted in a considerable amount of unused time for the MI counsellor and constituted a challenge in the implementation of the programme. The programme might be improved by adjusting the sessions more to the parents’ needs, e.g. conducting the second MI session for some parents over the telephone.

### Strengths and limitations

An important strength of the current study is that process evaluation has been done thoroughly, as emphasised by Waters and colleagues [20]. Many studies have neglected measuring process variables, including fidelity [26]. This is particularly true regarding the use of MI. In this study we monitored MI using a valid and reliable instrument, the MITI, used by skilled coders and tested for reliability in a university-based MI coding lab. Fidelity of all three intervention components was high, indicating that the programme was delivered as intended. Additional strengths were the cluster-randomised controlled design, the objective assessment of physical activity and anthropometry, and the fact that all the materials in the intervention were pre-tested. Further, this study collected follow-up data so that the effects six months after the intervention could be evaluated.

One limitation of the study is the self-report questionnaires, which might lead to under reporting of unhealthy behaviours and over reporting of healthy behaviours due to social desirability. In addition, potentially important aspects of dietary intake such as intake of sodium, saturated fat and fibre were not assessed. In addition, language problems might have been an issue for foreign-born parents since all the materials were in Swedish.

The effects of the intervention were small, although comparable to several other studies. Many outcomes were non-significant, although most of the dietary changes were in the desired direction. The relatively small effects could be due to several reasons: Either the intervention dose was too small or of too short duration, the instruments used to measure dietary intake were not sensitive enough, or the children’s health behaviours were at a good level to begin with. This was indicated by the fact that all of the children fulfilled the recommendation of 60 minutes of MVPA per day at baseline. Future studies should therefore preferably be carried out in areas with greater needs. The reason for choosing a relatively short intervention period of six months was the waiting-list control design of the study, where the control classes were promised to receive the intervention after the follow-up measurements were finished one year later. The short duration of the intervention could also have been of significance for the lack of intervention effect on parental self-efficacy. Studies that have been implemented for more than one year have shown the largest intervention effects [22].

Changes in behaviours and formation of habits can take a long time to implement and, further, vary between individuals [69]. Therefore, the programme might benefit from being repeated with reminders of the intervention components in some form every school year. In this study the changes in a child’s behaviour occurred mainly via the parent, which makes behaviour change even more complex. To summarise, a longer duration and continuous repetition of the programme might be needed in order to improve effectiveness of the programme and achieve a long-lasting impact.

One indication of lack of adverse effects of the programme was the prevalence of underweight, which remained unchanged from pre to post assessment. Further, the programme did not seem to increase health inequalities since there was no moderating effect of parental SES on the outcomes.

## Conclusion

The Healthy School Start is an appreciated programme and this study shows that it is possible, with relatively little effort, to influence vegetable intake in children and girls’ physical activity, by providing parental support. However, the positive effects were short-lived. Therefore, the programme probably needs to be prolonged and/or intensified in order to obtain stronger and sustainable effects. We believe that the inclusion of Motivational Interviewing as the main intervention component constitutes an important progress, because it implies parents participation, barrier identification, and prompt specific goal setting, all factors that have been identified as features of effective interventions [25]. This study may therefore be an important contribution to the further development of evidence-based parental support programmes to prevent overweight and obesity in children.

## Supporting Information

S1 Checklist CONSORT checklist. (DOC)

S1 Protocol Trial protocol. (DOCX)
