# Supplementary material for: Effectiveness of a Universal Parental Support Programme to Promote Healthy Dietary Habits and Physical Activity and to Prevent Overweight and Obesity in 6-Year-Old Children: The Healthy School Start Study, a Cluster-Randomised Controlled Trial
Source: PLoS One. 2015 Feb 13;10(2):e0116876. doi: 10.1371/journal.pone.0116876 (PMC4332680; doi:10.1371/journal.pone.0116876)
Supplement: S1 Protocol — (DOCX) [file pone.0116876.s002.docx]

**Clinical trial protocol translated into English**

**To the Regional Ethical Review Board in**: Stockholm

**Project title:** A Healthy School Start – parental support for healthy dietary and physical activity habits

**1. Information concerning the body principally responsible for the research etc.**

1:1 The responsible research body:

Intervention and implementation research, Department of Public Health Sciences, Karolinska Institutet, Box 17070, 104 62 Stockholm

1:2 Qualified representative of the body principally responsible for the research:

Lucie Laflamme, Head of department, Department of Public Health Sciences, Karolinska Institutet, 171 77 Stockholm

1:3 The researcher/s primarily responsible for conducting the project:

(principal contact person/s)

Dr Gisela Nyberg, Department of Public Health Sciences, Karolinska Institutet, Box 17070, 104 62 Stockholm, e-mail: [gisela.nyberg@ki.se](mailto:gisela.nyberg@ki.se), Telephone: +46-6009179

1:4 Arena:

9 schools in Nacka (outside Stockholm): Fisksätra, Lännbo, Strandpark, Björknäs, Igelboda, Vilan, Orminge, Skuru and Vittra in Saltsjöbo.

1:5 Other collaborators:

The project team: Associate Professor Liselotte Schäfer Elinder, Dr Gisela Nyberg, nutritionist, MSc, Elinor Sundblom. We are in the process of recruiting a person who will perform the Motivational Interviewing sessions. Health promotors from the municipality Eva Flodström, Eva Scharin and school physician Gunilla Myhrman.

**2. Information concerning the project**

2:1 Summary of the research project

Poor dietary habits and inadequate physical activity means that many children and adolescents run the risk of becoming overweight and developing disordered eating habits (irregular and/or restricted eating as well as body dissatisfaction). Childhood obesity is 3-5 times more common in families with low income and/or education compared to those with high. The prevalence of overweight is increasing and effective prevention measures are in demand. Data from Stockholm county from 2006-2007 indicates that the prevalence of overweight among children is probably stabilising or even decreasing in affluent areas, but the trend seen in more deprived areas is in the opposite direction. Strategies that promote healthy and regular eating habits and physical activity are believed to have the potential to prevent both overweight and disordered eating and promote healthy weight development among children and adolescents.

The school arena has the potential to even out health inequalities that may arise due to the different social, cultural and health behaviours of parents. Previous research has shown that prevention is more effective when community activities are complemented with home-based strategies. Given the uneven distribution of health inequalities and obesity which affects deprived groups disproportionally, the potential for prevention is great.

The home environment and parental behaviour have an important influence on children’s dietary habits. There is evidence that active parental encouragement and support is important for the development of good physical activity habits.

The school health care system is probably one of the best channels through which to reach parents concerning their children’s health development. At the health check-up conducted in the pre-school year, parents are present. This is an ideal opportunity to present a clear, consistent message to parents about health and attitudes. Our experience is that school health staff are very engaged and motivated regarding issues of dietary habits and physical activity, but that resources for preventive work is scarce. Staff are interested in learning more about the technique “motivational interviewing (MI)”. MI has been shown to be effective in helping people make changes to their dietary and physical activity habits in the clinical setting but this has yet to be thoroughly tested in the school setting.

We will study whether it is possible to improve children’s dietary and physical activity habits and weight development by conducting motivational interviews with parents through the school health care system and by the use of teacher-led classroom-based activities.

Aim

To develop and evaluate a parental support programme delivered in the school health care setting to promote healthy dietary and physical activity habits for parents of 6-year-old children.

Research questions:

How does motivational interviewing with parents, in combination with teacher-led classroom activities, affect children’s dietary habits, physical activity levels, weight development and parental self-efficacy to affect change?

Do children’s dietary habits, physical activity, body weight index (BMI) and parental self-efficacy differ by parental education level?

Research variables:

We aim to study 200 children in 16 pre-school classes in deprived areas in the municipality of Nacka. Half of the classes will be randomised to the intervention group and the other half to the control group.

The primary outcome will be the consumption frequency of certain indicator foods (fruit, vegetables and energy-dense foods) as assessed by questionnaire.

Using an accelerometer, physical activity will be evaluated as total step count per day, the average time in total physical activity over a week, the number of minutes at different intensities and time spent sedentary.

A qualitative study with parents and teachers will be conducted using focus groups and will be analysed using content analysis.

Parental self-efficacy (the ability to affect change and the belief that they are in control) will be evaluated using questions taken from the social cognitive theory.

Secondary outcomes include weight and length data obtained from the school health care journals. Waist circumference is also measured during the school health check-up. Body mass index (BMI) is calculated and overweight and obesity are identified using international cut-offs. In addition, BMIsds is calculated based on Swedish cut-offs.

Knowledge gains:

The school health care system lacks effective and evaluated tools for use in their communication with children and their parents. There is a demand for more resources to use in health check-ups but to the best of our knowledge, no studies have been conducted that show the effectiveness of such resources. The project could generate new ways for both teachers and school health care staff to work to promote health and reduce health inequalities among children. When the project is completed the method can be spread to other municipalities and regions.

School health care staff are very interested in learning about MI and becoming trained in its use. The results from our study are likely to be integrated into their usual routines. We hope that the study will show that with very meagre extra resources (for the MI), a relatively large effect on children’s lifestyle habits can be achieved. The pre-school year is a very suitable time to conduct a parental support programme as the children are still completely dependent on their parents and the classroom syllabus is very flexible. Only a few studies have examined parental support and children’s dietary and physical activity habits, and none has been carried out in Sweden that we know of.

2:2 What is/are the primary scientific question(s) forming the basis of the design of the

project?

Hypothesis: Motivational Interviewing in combination with teacher led classroom activities will promote healthy dietary and physical activity habits, weight development and parental self-efficacy to make changes.

2:3 State the results from relevant animal experiments (clinical trials):

N/A

2:4 Give an overview of the examination procedures used, data collection and the nature of

the data

The project will be carried out in collaboration with the school health care system in the municipality of Nacka, which has a long history of working with children and adolescent lifestyle behaviours. Nacka is a municipality with a relatively high median income and education level but also big differences in socioeconomic levels. Living conditions vary greatly between areas of the municipality and several health problems are a concern. One of these is childhood and adolescent overweight, which continues to increase. Our research group, which until recently belonged to Stockholm County Council, has a lot of experience with communicating health information and supporting local projects related to diet and physical activity in the county’s municipalities and organisations.

We aim to study 200 children in 16 pre-school classes in deprived areas in the municipality of Nacka. Half of the classes will be randomised to the intervention group and the other half to the control group.

The intervention consists of two motivational interviews three months apart for parents and teacher-led classroom activities for children.

During the spring of 2010, the principals of nine schools will be approached and invited to participate (Appendix 5:1). For the school to be included in the study, at least 30% of the families must be classified as deprived. Once the school has consented, the pre-school teachers will be invited. Parents will be informed (Appendix 5:1) at the regular parent-teacher meeting and a letter inviting them to participate will be sent at the beginning of the school term (Appendix 4). In order to reach the families the letter will be sent in tandem with the routine letter from the school health care. Parents consent to participate in the project by signing and returning the consent form (Appendix 4).

Baseline measurements of physical activity. Children will wear an accelerometer for a week. The physical activity is measured with an accelerometer which provides objective measures of different variables, such as total physical activity, sedentary time, total steps and time spent at different intensity levels. Accelerometry is a reliable method and has been used in many studies both nationally and internationally to measure children’s physical activity. The accelerometer will be given out by a member of the research team and collected after 7 days.

Baseline measurements of eating habits (intake frequency of fruit and vegetables and energy-dense products), physical activity habits, inactivity and sleep is assessed by means of a survey (Appendix 5:2). The survey questions are based in part on a validated English parent questionnaire. Parents' self-efficacy, ie, their confidence in their ability to be able to change their children's food and movement habits are measured by a questionnaire (Appendix 5:2). The questionnaire has been translated and modified for use in the area of food and physical activity based on a validated English questionnaire which measures parents' self-efficacy in parenting. Surveys are sent home to parents as soon as consent has been received by the researchers. The questionnaires are then handed in by the parents at the first motivational interview.

The intervention is planned to start in November. During the intervention, the teachers in the intervention classes follow a teacher's manual (Appendix 5:3) which is based on a brochure that has been designed as a result of a scientific review (www.folhalsoguiden.se / food). The brochure is designed to be educational, contains many illustrations and is written in simple Swedish (Appendix 5:4). It is based on the various themes of the review; 1. Good food and meals, 2. Movement/Physical Activity, 3. Candy, snacks, ice cream and soft drinks, 4. Fruits and Vegetables, 5. Advertising, TV and other media, and 6. Sleep and rest. The material contains facts and evidence-based advice to parents. The information in the brochure will form the basis for the future motivational interview. The brochure will be sent home to the families before the intervention starts, so that they have time to reflect on the content before the first motivational interview.

In the pre-school class the teacher will conduct 10 lessons (each 30 minutes in length) described in the teacher manual. Students will be assigned simple home exercises from a workbook on 7 occasions (Appendix 5:5). The purpose of the homework assignments is so that children remind their parents at home about the different themes that have been brought up at school.

During the routine health check-up (which assesses development progress, height, weight, immunisation status, etc.) in Nacka both a school physician and a school nurse are present together with the child and parents. Child's weight, height and waist circumference from the health check will be used in the study. In conjunction with the check-up, a person trained in motivational interviewing will conduct a 45-minute meeting with the parents. The conversation is based on the parents' needs and the content of the brochure. Goals for change will be formulated at the end of the meeting. Parents will be invited to a follow-up meeting after about three months, where they will discuss the changes that have been made. Parents will be given a diary to bring home in which they should record progress and goals met, every other week (Appendix 5:6). Text messages will be sent every other week as a reminder to fill in the diary. A text message will also be sent as a reminder of the scheduled MI meeting.

Our results will be based on questionnaires of the children's eating habits, and parents' self-efficacy assessed according to validated methods. Our conclusions about the children's physical activity will be based on objective measurement with accelerometers. Questionnaires, diaries and brochures will be pre-tested and adjusted if necessary before use in the study of a group of parents similar to the target audience. That the intervention is progressing as it should (compliance) will be checked by recording every twentieth motivational interview with the permission of the parents and (anonymously) coding them to ensure the quality of the motivational interviews. After each classroom lesson, the teachers will fill in a log/checklist so that we can check whether the teaching manual has been complied with and how much time the class has spent on each theme (intervention dose).

Randomisation will occur at school class level after baseline measurements have been made on all children. In the control classes the ordinary health check-up will be conducted as normal. No classroom activities or motivational interviewing will be offered. After the project is completed, all families in the control classes will be offered motivational talks and classes will have access to the brochures, teacher manual and workbook.

After the end of the intervention, after six months, the same measurements made at baseline will be made again. A lottery ticket will be offered to both the control and intervention families after the baseline measurements have been performed and another lottery ticket will be offered after the measurements at the end of the project have been made.

At the end of the intervention, 4 groups of parents (about 5-6 in each group) will participate in a focus group discussion with the aim of gaining an understanding the participants’ experience of the project. The discussion will be led by a researcher with extensive experience in focus group methodology. Focus group discussion is a method that is suitable for exploring people's experiences, perceptions, preferences or difficulties. The focus groups will be analysed using qualitative content analysis. Focus group discussions will also be conducted with teachers of the intervention classes to evaluate the classroom components and what obstacles and opportunities the teachers experienced.

A follow-up survey will be done a year after the start of the project with the same measurements at baseline and at the end of the project (questionnaires to parents about the child's eating habits, parental self-efficacy and measurement of the child's physical activity). The children will be weighed and measured again as part of the school health care system’s routines and the data will be collected by scientists from Karolinska Institutet.

An internal team of experts has collaborated to develop the materials in the project. A teachers' group of six teachers have helped to develop the teacher's guide and workbook for children. A steering committee comprising members from Karolinska Institutet, school health and public health planners from Nacka meet once a month to prepare and plan the project.

2:5 Describe how collected biological material is to be stored in a biobank:

N/A

2:6 Funding of the study:

This study is funded by the Public Health Fund, Stockholm County Council and the Signhild Engkvist Foundation.

2:7 Documentation, data protection and record-keeping:

All names and personal data will be replaced with a code number in order to de-identify participants. These code lists will be kept locked up. Lists of personal numbers, names, and addresses will be kept locked in a filing cabinet at Karolinska Institutet. Questionnaire and accelerometer data kept locked in a filing cabinet at Karolinska Institutet. Data on weight and height measurements will be collected by the project team from the school nurse and will be kept under lock. The de-identified data will be entered into a data file by the project team. The material that will be processed and analysed is guaranteed confidentiality. Throughout the project, the principal researcher and participating researchers will have access to the material. After completion of the project all collected material will be archived according to the prevailing rules at Karolinska Institutet.

2:8 Describe previous experience (your own and/or others’) of the procedure, technique or

treatment used:

The methods used in the present research are scientifically proven and established in the scientific community. The researchers in the project have collective experience and knowledge of these methods and have previously produced a number of reports, theses and articles. The project team has good knowledge and experience of working with questionnaires, accelerometers, focus groups, motivational interviewing and working with children in school-based projects. The project group has previously published reports and scientific articles that essentially apply the same methodologies chosen for this project. The principal investigator is, in consultation with a statistician, responsible for the statistical analysis and has extensive experience of school-based studies and analysis of children's physical activity, diet and weight development.

The concepts of weight development, overweight and obesity will not be mentioned to the children. The project focuses on delivering a positive health message which emphasises that good food and physical activity habits leads to children having more energy in school and getting a healthy start to school life.

No risk of complications for any of the methods proposed in this project have been identified through previous studies or in the existing literature.

**3. Information about the research participants**

3:1 How are research participants chosen?

Participants will be recruited from schools in Nacka’s more deprived areas: Fisksätra, Orminge and Älta, where the prevalence of overweight and obesity is higher than in other parts of the municipality. Schools in Nacka, where at least 30% of families are considered deprived (inclusion criteria) will be invited to participate in the project. The research group has previously collaborated with Nacka Municipality which has a lot of experience in diet and health promotion work focusing on children and adolescents. Where both parents do not understand Swedish, i.e. if they need an interpreter in order to participate in the motivational interview, the family will be excluded (exclusion criteria). We hope in the future to be able to offer the material translated into several languages and an interpreter to help with motivational session. In this study, however, it is not practical or feasible.

The public health planner from Nacka Municipality will contact principals at the included schools and arrange a time for an initial meeting with the project team. All pre-school classes will be invited to participate. Principals and teachers will be informed by the project team. The project team will inform parents at the annual parent meeting. The parents will then be contacted by letter in tandem with the usual letter from the school health service. Once parents give their consent they are participating in the project. Approximately 14-16 classes from nine different schools will be randomised to either the intervention or control group.

3:2 State the relationship between the researchers/leader of the research and those participating in the research:

Evaluator

3:3 State the statistical foundation with respect to the size of the population(s) and/or

material(s) studied:

Primary outcome is physical activity measured with accelerometry. A “sample size calculator for cluster randomised trials” was used for calculation (Campbell et al, 2004). The estimated sample size was calculated for a two-sided test with the significance level of 0.05 and power was set to 90%. The calculation shows that 12 school classes with a participation rate of 60% in each class, approximately 144 children in total, are needed to detect a 20 % significant increase of physical activity steps between the intervention and control groups. Another outcome in physical activity such as a decrease in sedentary time needs fewer children. It was decided to randomise 200 families in total (100 families in the intervention group and 100 families in the control group) to have a margin for drop-outs and withdrawals.

3:4 State if participants in the research may be included in several studies, either

simultaneously or in another study or other studies closely linked to this one. If so, what

kind of research?

No

3:5 What insurance cover is there for research participants taking part in the project?

The participants are insured within the schools.

3:6 What financial remuneration or other benefits are participants in the research entitled

to and when is this to be paid?

Two lottery tickets

**4. Information and consent**

4:1 The procedure involved and the content of the *information* that is given when subjects

are asked to participate in the research:

The public health planner from Nacka Municipality will contact principals at the included schools and arrange a time for an initial meeting with the project team. All pre-school classes will be invited to participate. Principals and teachers will be informed by the project team. The project team will inform parents at the annual parent meeting during the spring and autumn of 2010. The parents will then be contacted by letter containing information and a consent form (Appendix 4) in tandem with the usual letter from the school health service at the start of the autumn term 2010. The participants will be informed about the study and that participation is voluntary and that they can withdraw at any point. Many families have an immigrant background so the information will be formulated in simple Swedish to facilitate their understanding and consideration of the information.

4:2 How is *consent* to be obtained and from whom?

Parents of children in pre-school classes in the selected schools will be contacted by letter signed by the project team at Karolinska Institutet, the school physician and public health planner from Nacka Municipality. The letter will inform them about the study's purpose and procedures, and the type of data that will be collected and how the data will be processed. Parents and children will be informed that participation is voluntary and that they can at any time choose to withdraw. The consent form is returned signed by a parent with a pre-paid envelope to the research team who registers either the consent or that they have declined to participate.

Forms will be kept locked in a filing cabinet at Karolinska Institutet.

**5. Considerations in the light of research ethics**

5:1 Describe the risks that participation might entail:

The risk of privacy violation and/or discomfort is judged as minor as is the risk of complications. The accelerometers are worn on the hip attached to a belt and will not involve any discomfort for the children.
The motivational interview will be performed by a person with documented MI skills and will therefore not present a risk of discomfort or pressure.
During the focus group discussions, the various issues will not be intrusive. The moderator and assistant of the discussions will be responsible for ensuring no one is put in a vulnerable situation.

5:2 Describe the predictable benefit for the people participating in the research who are

part of the project (applies especially to treatment):

The project aims to prevent both overweight and disordered eating behavior and thus promote normal weight gain in the children who participate in the project. Many of the families participating in the project are deprived families where obesity is 3-5 times more common compared to families with more resources. Given the uneven distribution of health and obesity which negatively affects deprived groups there is great potential for prevention for the children involved in the project.
The benefits of the project for the parents is that they can get a better understanding of good food and physical activity habits for themselves and their children and may be bolstered in their confidence to bring about change and the ability to set limits.

5:3 and 5:4 In a broader perspective, identify and specify which ethical problems, such as risk versus benefit, can arise as a part of or as a result of the project:

The potential risks are deemed to be very small compared to the benefits in terms of improved health of children and families.
There is always a risk with this type of project in which a group might feel singled out (deprived). This must be weighed against benefits in terms of improving health among these children.
The risk that parents could be made to feel like bad parents is considered small because the person who will conduct the motivational interviews has documented experience.
Overall, the benefits outweigh the risks.

**6. Presenting the results**

6:1 How are both the entity principally responsible for the research and research

collaborators guaranteed access to data (to be stated when the research is commissioned) and who is responsible for processing data and writing reports?

The principal investigator, Gisela Nyberg, will be responsible for data collection, data processing and report writing. Researchers at Karolinska Institutet will have access to data. Results will be published jointly by the research team.

6:2 How will the results be made publicly available? Will the study be sent for publishing in

a journal or published in some other manner?

Results will be made public via the publication of reports and in scientific journals in 2011 and 2012. Results will also be presented at national and international congresses in 2011 and 2012. A manual will be developed in 2011 and 2012 for the school health services regarding how schools can develop parental support for promoting healthy food and physical activity habits.

6:3 In what manner will the right to integrity of those participating in the research be

guaranteed when the material is made public or is published?

Reporting is completely anonymous which means that no names will be listed and that participating individuals cannot otherwise be identified. The results are reported on group level. Name and address information is encoded with a serial number and stored in a locked cupboard, separate from the data file, during and after the study.

**7. Reporting the financial circumstances and dependencies**

7:1 When the research is commissioned:

N/A

7:2 Give an account of any financial agreements with a responsible body or any other financiers

(name, amount):

N/A

7:3 Give an account of the interests of the responsible research body, the principal researcher and of participating researchers:

N/A

**8. Signatories**

Please see original document in Swedish

**9. List of annexes**

Please see original document in Swedish
